# Aluminum silicide microparticles transformed from aluminum thin films by hypoeutectic interdiffusion

**DOI:** 10.1186/1556-276X-9-312

**Published:** 2014-06-21

**Authors:** Jin-Seo Noh

**Affiliations:** 1 Department of Nano-Physics, Gachon University, 1342 Seongnamdaero, Sujeong-gu, Seongnam-si, Gyeonggi-do 461-701, South Korea

**Keywords:** Aluminum film, Al-Si alloys, Microparticles, Hypoeutectic temperatures

## Abstract

Aluminum silicide microparticles with oxidized rough surfaces were formed on Si substrates through a spontaneous granulation process of Al films. This microparticle formation was caused by interdiffusion of Al and Si atoms at hypoeutectic temperatures of Al-Si systems, which was driven by compressive stress stored in Al films. The size, density, and the composition of the microparticles could be controlled by adjusting the annealing temperature, time, and the film thickness. High-density microparticles of a size around 10 μm and with an atomic ratio of Si/Al of approximately 0.8 were obtained when a 90-nm-thick Al film on Si substrate was annealed for 9 h at 550°C. The microparticle formation resulted in a rapid increase of the sheet resistance, which is a consequence of substantial consumption of Al film. This simple route to size- and composition-controllable microparticle formation may lay a foundation stone for the thermoelectric study on Al-Si alloy-based heterogeneous systems.

## Background

Thermoelectric energy generation, by which waste heat is converted into electricity, has emerged as one of key energy renewal technologies since a huge amount of world energy is wasted in the form of heat [1-3]. The performance of a thermoelectric material is determined cooperatively by the Seebeck coefficient (*S*), thermal conductivity (*κ*), and the electrical conductivity (*σ*) of the material [4]. Unfortunately, these three parameters have some intercorrelations in bulk, limiting the thermoelectric performance of a bulk material [5]. In this regard, one-dimensional (1D) nanowires have been highlighted, where a combination of quantum confinement effect and phonon boundary scattering drastically enhances the thermoelectric performance [6-8]. However, the controlled growth of thermoelectric nanowires and the reproducible fabrication of energy conversion modules based on them should be further demonstrated. Two-dimensional (2D) thin films have the superiority in terms of the ease of material and module fabrication and the reproducibility of the thermoelectric performance.

The best thermoelectric materials reported to date include Bi_2_Te_3_[9], AgPb_m_SbTe_2+m_[10], and In_4_Se_3−δ_[11]. These materials, however, contain chalcogens (Se, Te), heavy metals (Pb, Sb), and rare metals (Bi, In), all of which are expected to restrict the widespread use of these materials. Recently, it has been demonstrated that even a conventional semiconductor, silicon (Si), can exhibit thermoelectric performance by adopting nanostructures such as nanowires [12], nanomeshes [13], and holey thin films [14]. Although Si has a high *S* of 440 μV/K, its electrical conductivity is poor (0.01 ~ 0.1 S/cm) [15]. Thus, alloying Si with a good metal could lead to the improved thermoelectric performance. Aluminum (Al) is a typical good metal that has the advantages of high electrical conductivity (approximately 3.5 × 10^5^ S/cm) [16], light weight, and low cost. Despite the expected high electrical conductivity, the thermal conductivity of Si-Al alloys may be still high due to the large thermal conductivities of the constituents: *κ*_Al_ = 210 ~ 250 W/m K and *κ*_Si_ = 149 W/m K at room temperature [17]. The thermal conductivity of the alloy can be reduced by introducing nano- or microstructures on the alloy film. For this reason, embodying nano- or microstructures on Al-Si alloy films is a critical prerequisite for the study of thermoelectric performance of heterostructures made of Al-Si alloys.

In this work, aluminum silicide microparticles were formed from Al thin films on Si substrates through self-granulation. This process resulted from solid-state interdiffusion of Al and Si at hypoeutectic temperatures, which was activated by compressive stress stored in the films. This stress-induced granulation technique is a facile route to the composition-controlled microparticle formation with no need of lithography, template, and chemical precursor.

## Methods

**Figure 1:**
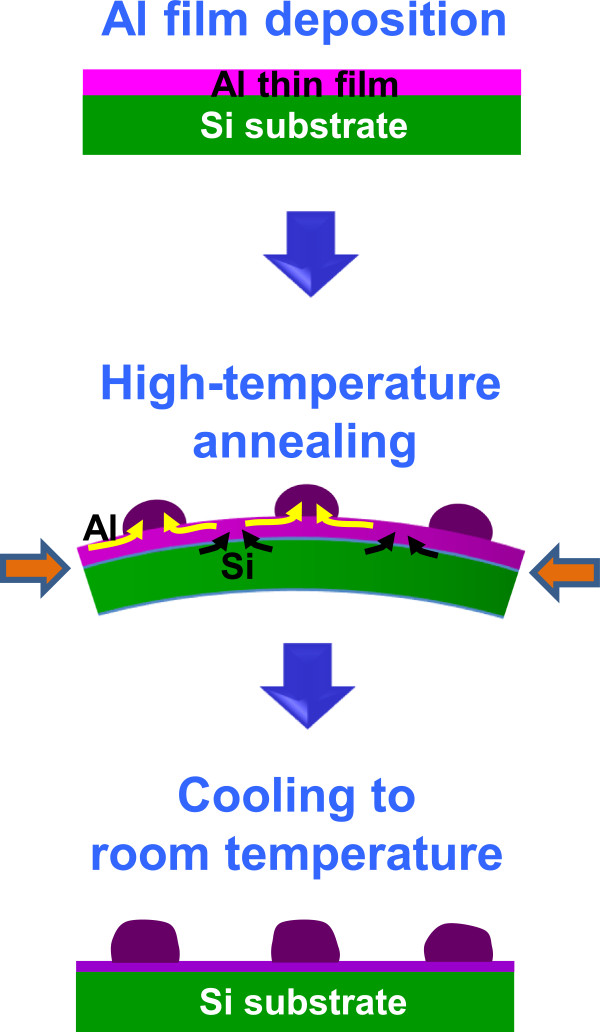
**Al-Si microparticle formation from Al thin film on Si substrate.** Step 1: Al thin film deposition by RF sputtering, step 2: high-temperature annealing, and step 3: cooling down to room temperature. In step 2, compressive stress is stored in Al film due to the difference in thermal expansions of Al film and Si substrate, and interdiffusion of Al and Si atoms is accelerated to relieve this stress, leading to granulation. As a consequence of granulation, the original Al film is almost exhausted.

Figure 1 schematically shows the procedures of Al-Si microparticle formation on Al thin films. First, Al thin films were deposited onto (100) Si substrate by radio frequency (RF) magnetron sputtering at room temperature. The base pressure of the chamber was about 10^−7^ Torr and the process pressure was 14 mTorr (Ar flow rate, 16 sccm). Controlling the RF power (50, 100 W) and the sputtering time (10, 20 min), Al films with varying thicknesses (15, 40, 90 nm) were prepared. In the next step, the Al films on Si substrates were subjected to thermal annealing inside a quartz tube that was connected to vacuum pumps. The pressure inside the tube showed a change within a range of 2 × 10^−5^ to 8.7 × 10^−6^ Torr during annealing. Annealing temperature (400°C, 550°C) and time (3, 6, 9 h) were controlled as a variable. Due to the higher thermal expansion coefficient of Al (23.1 × 10^−6^/K) than that of Si (3 × 10^−6^/K), the system is slightly bent and compressive stress is stored in Al film. To relieve the compressive stress, diffusional surface flow of Al atoms and outward diffusion of Si atoms occur at elevated temperatures, leading to the formation of Al-Si microparticles. This process is similar to the on-film formation of nanowire growth (OFF-ON) previously reported [18], but microparticles rather than nanowires are formed as the diffusivities of Al and Si are much larger than those materials used in OFF-ON [19,20]. Finally, Al films with Al-Si microparticles on Si substrates were naturally cooled down to room temperature. During this cooling step, vacuum pumps were not operated so that surface oxidation occurs.

The surface morphology of Al films on Si substrates was examined first at micrometer scale using a laser-scanning microscope (LSM, Olympus CLS 4000; Olympus Corporation, Tokyo, Japan). This was conducted on both as-deposited films and heat-treated films. More in-depth morphology study was performed employing a field emission scanning electron microscope (FE-SEM, Hitachi S4300; Hitachi High-Tech, Tokyo, Japan) equipped with energy-dispersive x-ray spectrometer (EDX). The electron acceleration voltage was set at 15 kV. Atomic force microscopy (AFM, Veeco Metrology, Santa Barbara, CA, USA) was also utilized for nanoscale analysis and step height measurement. The structure and the composition of heat-treated samples were analyzed using x-ray diffraction (XRD, Philips X’Pert PW3040; Koninklijke Philips N.V., Amsterdam, Netherlands). In addition, sheet resistances of untreated and heat-treated samples were measured employing a standard four-probe method and their correlation with structural transformation was studied.

## Results and discussion

**Figure 2:**
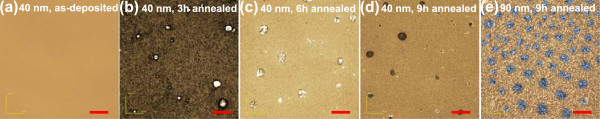
**LSM images of an as-deposited and annealed Al films on Si substrate. (a)** As-deposited film. Samples annealed at 550°C: **(b)** 3 h, **(c)** 6 h, **(d and e)** 9 h. **(a to d)** 40-nm-thick Al films and **(e)** 90-nm-thick Al film. Scale bars 20 μm.

Figure 2 shows LSM images of an as-deposited Al film and samples annealed for different durations at 550°C. The surface of as-deposited Al film is smooth, as seen in Figure 2a. When the 40-nm-thick Al film on Si substrate is annealed for 3 h, particles with a size distribution of 0.3 to 7 μm start to form on the surface. This indicates that Al atomic flow is activated at this condition and forms randomly distributed seeds of Al particles. Prolonging the annealing time to 6 h, small particles disappear and large particles with more size uniformity are left behind, which may result from the agglomeration of small particles. The particle size is in general larger than 5 μm. At a longer annealing time of 9 h, the particle size distribution is similar to the case of 6 h annealing, but small pit-like nonuniform structures are observed in the film, presumably originating from local Al deficiency and Si inflow from the substrate. It is inferred that Si's outward diffusion and its mixing with Al atoms are the reasons why the color of the particles in Figure 2d is dissimilar to that in Figure 2c. If it is the real case, the microparticles should not be pure Al, but Al-Si alloys. The density and the average size of particles are apparently found to increase as the Al film thickness increases, as demonstrated in Figure 2e. This is because the Al film plays as a major source material nourishing the microparticles and the particles become bigger and denser at the expense of the film. For the 90-nm-thick Al film, the density of the particles is calculated to be 2,500 to 5,560 mm^−2^ and the particle size reaches up to 13 μm. This spontaneous granulation was rarely observed when an Al film on Si substrate was annealed at 400°C, justifying that the microparticle formation is a process caused by atomic diffusion.

**Figure 3:**
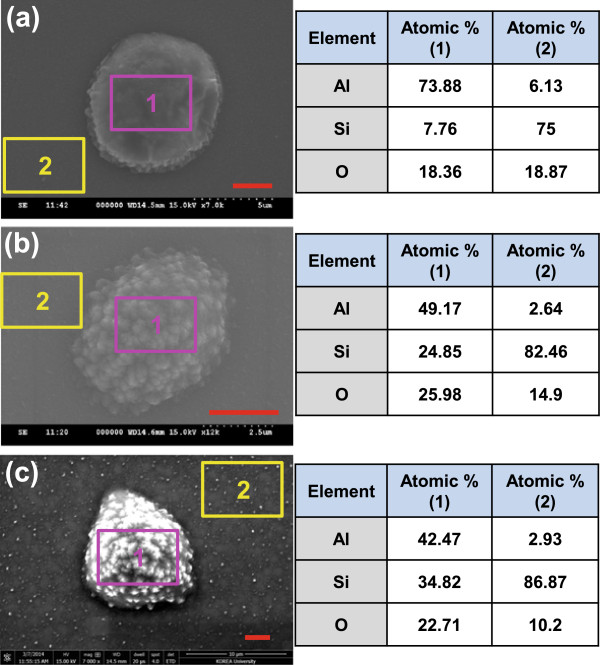
**SEM images of microparticles.** SEM images of microparticles transformed through **(a)** 6 h annealing and **(b)** 9 h annealing of a 40-nm-thick Al film and **(c)** 9 h annealing of a 90-nm-thick Al film on Si substrate. Annealing temperature was set at 550°C. Scale bars 2.5 μm. EDX element analysis results are also presented for the particle area (notated as ‘1’) and the rest (notated as ‘2’), respectively.

The detailed structure and the composition of microparticles were analyzed using SEM. Figure 2 exhibits top view SEM images of three samples corresponding to Figure 2c,d,e, respectively. The general shape of the microparticles looks like a distorted hemispheroid with rough surface. It was observed from tilted views that the out-of-plane height relative to in-plane diameter becomes larger with an increase in the average particle size (not shown). From the point of composition, the microparticles are not pure Si, but Al-Si alloys, as deduced from the previous LSM images, with some amount of oxygen. The observed oxygen content is considered to stem from the surface oxidation of the microparticles during cooling and in storage [21]. Interestingly, the composition of the microparticles changes by controlling the annealing time and the film thickness. Comparing Figure 3a (6 h annealed) and Figure 3b (9 h annealed), the atomic ratio of Si to Al of the microparticle formed through 9 h annealing (50.5%) is much larger than that of the microparticle which underwent 6 h annealing (10.5%). Taking into account that the annealing temperature (550°C) of the present study is lower than the eutectic temperature (577°C) of Al-Si systems and the Si solubility in Al crystal is only about 1.4 at. % at 550°C [22], the measured large Si concentrations reflect solid-state interdiffusion of Al and Si atoms facilitated by compressive stress that is developed by larger expansion of Al film than Si substrate during annealing (see the middle panel of Figure 1) [23,24]. It is speculated that more mobile Al atoms move first over the surface or through grain boundaries to agglomerate, leaving behind a lot of vacancies. These vacancies in Al film may accelerate outward diffusion of Si atoms and direct Si atomic flow to Al granules to finally form Al-Si alloys. In addition, since the surface energy of Si (100) plane is relatively high (2.13 J/m^2^) [25], Si atoms are prone to diffuse into a foreign material at elevated temperatures to reduce the surface energy. This hypoeutectic interdiffusion progresses further as the annealing time is made longer. The atomic ratio of Si/Al rises to 82% for a microparticle from the 90-nm-thick film, as shown in Figure 3c. This may be because a larger volume of Al vacancies in Al film absorbs more Si atoms from the substrate. As a consequence of Al-Si microparticle formation, the majority of the original Al film is exhausted as seen in Figure 3b,c. Interestingly, it is found from Figure 3c that the residual Al film resembles the network structures of narrow channels.

**Figure 4:**
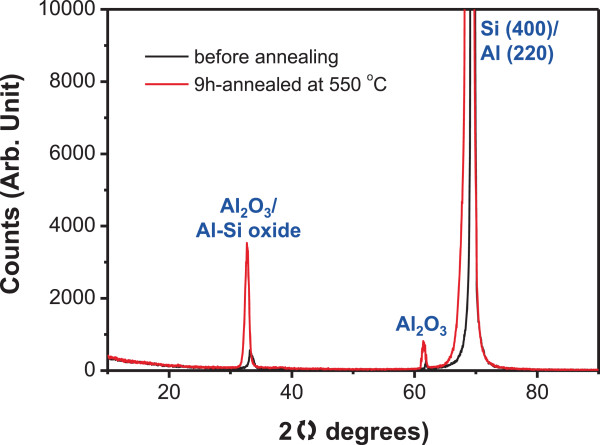
**XRD patterns of a 90-nm-thick Al film on Si substrate before and after annealing.** Samples annealed for 9 h at 550°C.

The composition and the crystal structure of both untreated and heat-treated Al films on the Si substrate were further analyzed using XRD. Figure 4 shows XRD patterns of 90-nm-thick Al films before and after annealing. For both samples, three major peaks are sharp, representing the samples are crystalline irrespective of heat treatment. The overwhelming peak of 68° to 69° is assigned to Si (400). Al (220) peak that usually appears around 66° is presumed to be superposed with the Si (400) peak. The other two peaks observed at approximately 33° and 62° are related to Al_2_O_3_ or Al-Si oxide. The peak intensities of a 9-h annealed sample are far larger than those of the untreated sample at those 2*θ* angles, particularly at approximately 33°. Recollecting that the XRD patterns resulted from a combination of microparticles and their surrounding areas, the microparticles are likely to be Al-Si oxide while the areas surrounding the microparticles may be Al_2_O_3_, which is in general consistent with the SEM-EDX results discussed above. Al_2_O_3_ peaks observed even for the untreated sample may originate from the surface oxidation of Al film at ambient condition.

**Figure 5:**
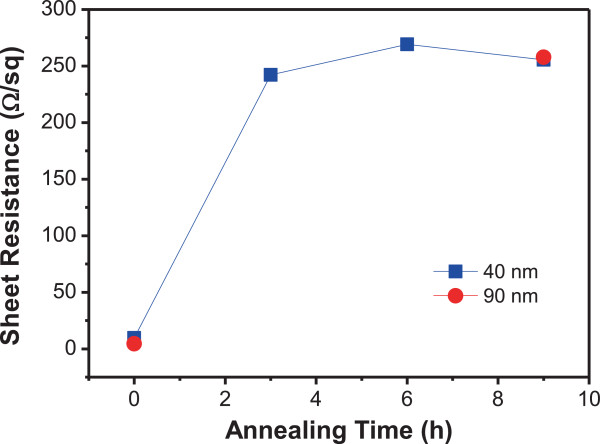
**Sheet resistance of a 40-nm-thick Al film on Si substrate as a function of annealing time.** Annealing temperature was fixed at 550°C. The sheet resistance rapidly increases after 3 h annealing and then almost saturates. For comparison, sheet resistances of a 90-nm-thick Al film before and after 9 h annealing are also plotted.

Figure 5 shows the variation of sheet resistance against annealing time for a 40-nm-thick Al film on Si substrate. For comparison, the sheet resistances of an untreated and a 9-h annealed 90-nm-thick Al films are also plotted. The distribution of sheet resistances at each data point was less than 3% around the average value, leading to the overlap of error bars with the symbols representing the average. The sheet resistance of the sample increases by approximately 25 times after 3 h annealing at 550°C. This is an indicator that spontaneous granulation has significantly progressed and the initial Al film was substantially consumed in the middle of the process (see the particles of a variety of sizes in Figure 2b). Although the sheet resistance of the sample is determined by the combined effects of particles and residual film, it is reasonable to think that the residual film is a dominant player due to the small size of the particles. Raising the annealing time further, the sheet resistance slightly increases, then almost saturates at about 260 Ω/sq, which corresponds to a 27-fold increase from the initial value. The slight increase of the sheet resistance may be caused by the further granulation and Al-Si alloying. The sheet resistances of a 40-nm-thick and a 90-nm-thick Al films after 9 h annealing are close to each other, reflecting that microparticle formation accompanying Al film consumption has maturely taken place in both samples. The resistivity (*ρ*) of the untreated Al films was (3.8 to 4.1) × 10^−7^ Ω m when calculated using a simple relation, *ρ* = *R*_s_ × *t*, where *R*_s_ and *t* are the sheet resistance and the thickness of the film, respectively. This calculated value is more than an order of magnitude larger than the literature value [(2.65 to 2.82) × 10^−8^ Ω m] [16,26], which is attributable to the presence of Al_2_O_3_ layer on the surface of Al films. The surface-oxidized microparticles of Al-Si alloys and the channel network structures of the surface-oxidized Al films are expected to cooperatively suppress the thermal conduction through the heterogeneous systems, resulting in the improved thermoelectric performance.

## Conclusions

Al thin films of 20 to 90 nm in thickness were deposited on Si (100) substrates by RF sputtering. Al films on Si were vacuum-annealed for 3 to 9 h at 400°C and 550°C, which are lower than the eutectic temperature of Al-Si systems. At hypoeutectic temperatures, compressive stress is developed in the films due to the larger thermal expansion of Al film than Si substrate, and this stress facilitates diffusional flow of Al atoms followed by outward diffusion of Si atoms. This interdiffusion of Al and Si atoms resulted in Al-Si alloy microparticles with rough surfaces, which were spontaneously granulated at the cost of the initial Al film. The density, average size, and the composition of the microparticles could be controlled by adjusting several parameters such as the film thickness, annealing temperature, and time. The surfaces of the microparticles and the residual Al film turned out to be oxidized, presumably during cooling and at ambient condition. As a consequence of the microparticle formation, the sheet resistance of Al film on Si substrate increased 27-fold after 9 h annealing at 550°C. This simple technique for the formation of Al-Si microparticles on Si substrate would be a stepping stone for the systematic study of the thermoelectric performance of heterogeneous systems based on Al-Si alloys.

## Competing interests

The author declares that he has no competing interests.
